# What Evidence is Available on Rapid Response Systems Across Europe? Findings From a Scoping Review

**DOI:** 10.1111/nicc.70217

**Published:** 2025-10-23

**Authors:** Sara Zamò, Federico Fonda, Alvisa Palese, Alessandro Galazzi

**Affiliations:** ^1^ Department of Medicine University of Udine Udine Italy; ^2^ Department of Medicine and Surgery LUM University Casamassima (Bari) Italy

**Keywords:** cardiac arrest team, hospital rapid response team, in hospital emergency, scoping review

## Abstract

**Background:**

Rapid Response Systems (RRS) aim to implement systematic processes for the early detection and management of emergencies in hospitals. Exploring the current characteristics and organisation of RRS in Europe and evaluating clinical outcomes may help identify areas for improvement.

**Aim:**

To provide an overview of the studies conducted in Europe, summarising the characteristics of RRS, their organisational structure and the clinical outcomes documented to date.

**Study Design:**

A scoping review was conducted according to the framework of Levac and reported according to the Preferred Reporting Items for Systematic Reviews and Meta‐Analyses extension for Scoping Reviews (PRISMA‐ScR) guidelines. The review protocol was registered with Open Science Framework (OSF); CINAHL, Cochrane Library, PubMed and Scopus were then searched. Quantitative primary studies about RRS conducted in Europe and published in English were included.

**Results:**

Twenty‐three studies were found, of which 18 (78.3%) were published in the last 10 years. Eleven (47.8%) aimed to describe the function and activities of RRS, while 12 (52.2%) analysed patient outcomes and two (8.7%) the effectiveness, feasibility and costs of RRS. Heterogeneous organisational patterns of RRS emerged, including terminologies (Medical Emergency Team [MET], Rapid Response Team [RRT], Cardiac Arrest Team [CAT], Critical Care Outreach [CCO]), team composition, availability, activation systems and activities.

**Conclusions:**

The number of RRS studies is increasing, especially to compare their organisational structure under heterogeneous terminologies. Future research should investigate the outcome variations between different RRS structures. In addition, guidelines that standardise the minimum data set in the field of RRS by also introducing registries could increase consistency between European practices and studies.

**Relevance to Clinical Practise:**

Knowledge of the current situation of RRS in Europe can serve as a basis for policies and benchmark models to promote better patient care.

**Study Registration:**

This scoping review was registered on Open Science Framework (OSF) on 3 August 2024 (https://osf.io/ekb95; https://doi.org/10.17605/OSF.IO/EKB95).

**Impact Statements**

- What is known about the topic
  ○ Hospital emergencies are a common problem that could potentially be prevented.
  ○ Rapid Response Systems are the services that have been created worldwide to deal with in‐hospital emergencies.
- What this paper adds
  ○ Rapid Response Systems in Europe have heterogeneous characteristics in terms of denomination, composition, availability, activation and activities of the teams.
  ○ The available studies mainly describe the function and activities of Rapid Response Systems rather than patient outcomes for patients.

## 1 Background

Clinical deterioration, defined as a transition to a worse clinical condition that increases the risk of morbidity or death, occurs in 3%–9% of hospitalised patients [1]. Around half of hospitalised emergencies (e.g., cardiac arrest [CA]) are considered preventable, as patients usually show the first signs of clinical deterioration in the last few hours before it occurs [2]. There is evidence that the incidence of in‐hospital CA is 1–6 per 1000 hospital admissions. However, 59.4% of patients have at least one abnormal vital sign between 1 and 4 h before CA, and 13.4% have at least one severely abnormal vital sign [3]. Because patients require urgent resources that are typically available in the emergency department and intensive care unit (ICU) but are not immediately accessible on general wards [4], in‐hospital emergency systems, such as Rapid Response Systems (RRS), have been introduced since 1990 [5] and subsequently recommended [6, 7].

The RRS aims to ensure systematic processes to prevent delays in recognising and responding to clinical deterioration. It applies the principles of early detection through predefined indicators of deterioration and the appropriate response [8]. Their role is to provide a safety net for patients who become critical and where an imbalance between needs and available resources may lead to further deterioration [4]. The term RRS is used to describe the entire system and not just its various components. This includes the afferent limb, that is, the tools to identify at‐risk patients and call for help, and the efferent limb, that is, a healthcare team that responds to requests for help and must be available 24/7; there is also an administrative and data analysis component [4]. There are different types of RRS, depending on the composition and leadership of the team. Teams that include physicians are usually coordinated by physicians and are referred to as Medical Emergency Team (MET), while teams that are primarily coordinated by nurses are referred to as Rapid Response Team (RRT) [9]. There are also teams that focus exclusively on CA, known as Cardiac Arrest Teams (CAT) [10]. Critical Care Outreach (CCO) is a service led by intensive care nurses that supports ward nurses in the care of potentially unstable patients [11].

RRS have been summarised in their main models in Australia and the United States of America (USA) as complex systems that require appropriate organisation and training for their effective implementation. Their characteristics in terms of designation, team members and associated outcomes are variable due to different delivery patterns, with an increased capacity to recognise patients at risk of deterioration in hospital and have been documented mainly in Australia [8, 12]. Individual studies have been conducted across Europe that have not yet been mapped. Therefore, the main aim of this scoping review was to summarise studies on RRS across Europe to describe the state of research and practice to inform policy and benchmark models in different countries and encourage further progress.

## 2 Aim

The aim was to map studies conducted throughout Europe, summarising the key features of RRS, its organisational patterns and the clinical outcomes as documented to date.

## 3 Design and Methods

### 3.1 Study Design

A scoping review was conducted according to the framework of Levac et al. [13] and according to the reporting guidelines of the Preferred Reporting Items for Systematic reviews and Meta‐Analyses extension for Scoping Reviews (PRISMA‐ScR) [14] (Table S1). The review protocol was registered (https://doi.org/10.17605/OSF.IO/EKB95) in the Open Science Framework (OSF) on 3 August 2024 [15].

### 3.2 Research Questions

The research questions were: (a) Which RRSs have been documented in studies in Europe to date? (b) What are the main characteristics of European RRSs in terms of composition, implementation, staff education, activities and alert systems? and (c) What patient outcomes (e.g., in‐hospital mortality) have been documented to date?

### 3.3 Inclusion and Exclusion Criteria

All primary quantitative studies of RRS understood as in‐hospital emergency response teams as studied across Europe and published in English at any time were eligible. Studies of RRS as response systems for out‐of‐hospital emergencies, qualitative or secondary studies (i.e., literature reviews), grey literature, protocols or retracted studies were all excluded.

### 3.4 Search Strategy

Firstly, different available terminologies such as MET, RRT [9], CAT [10] and CCO [11] were analysed. Then the Cumulative Index to Nursing and Allied Health Literature (CINAHL), Cochrane Library, PubMed and Scopus databases were searched using the following search terms: “Hospital Rapid Response Team” OR “Rapid Response System” OR “Rapid Response Team” OR “Medical Emergency Team” OR “Outreach Critical Team” OR “Cardiac Arrest Team”. The specific search term with its adaptation/restrictions per database is listed in Table S2.

### 3.5 Selection of Studies

**FIGURE 1:**
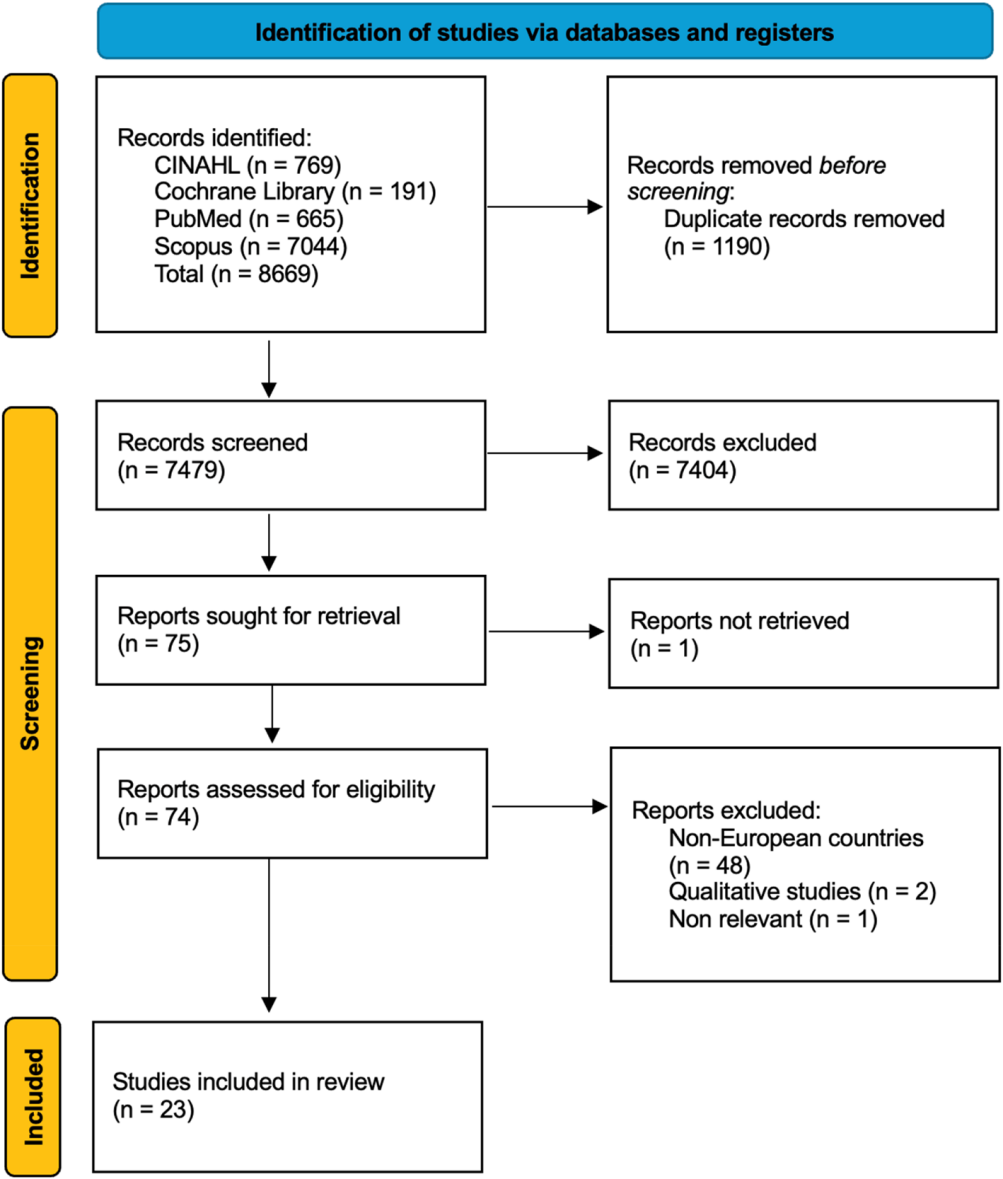
Flow diagram of the study selection process. CINAHL, Cumulative Index to Nursing and Allied Health Literature.

The search was carried out on 5 August 2024. The combination of data from the included databases yielded 8669 results, of which 1190 were removed before screening with the Rayyan software because they were duplicates. Screening was performed independently by two authors using the Rayyan software on the basis of titles and abstracts. In case of discrepancies, a third author was consulted. 7404 articles were excluded as they were not relevant to the research questions. Of the 75 articles whose full text was searched, one could not be found, and the corresponding author was contacted without success. The full text of the remaining 74 articles was then analysed: 48 of these articles were excluded because they were not conducted in Europe, two articles were excluded as qualitative studies, and one because it did not meet the inclusion criteria. A total of 23 studies were included, as shown in Figure 1 [16].

### 3.6 Data Extraction and Synthesis

The following data were extracted from all studies: (1) author, year of publication, journal (2) purpose(s) of the study (3) study design, period of data collection, setting and country (4) who can activate the RRT, the telephone number, the criteria for activating the system and the means of communication used (5) composition of the team, its stationing location and whether the team is dedicated exclusively or not (6) why it is activated, in which departments, by whom and the time (of arrival and stay on site) (7) the main activities performed and the documentation required (8) the patients' outcomes documented in the study.

Data extraction was performed by two authors, with a third author being contacted in case of discrepancies.

The extracted data were then described in a narrative fashion according to the research questions [17]: the main characteristics of the available studies, the documented RRS organisational patterns, and the patient outcomes, where reported, were summarised.

## 4 Results

### 4.1 Characteristics of the Studies

As indicated in Table 1 (and detailed in Table S3), the included studies [18, 19, 20, 21, 22, 23, 24, 25, 26, 27, 28, 29, 30, 31, 32, 33, 34, 35, 36, 37, 38, 39] were published between 2004 and 2024, of which 19 (82.6%) were published after 2011. They were conducted in 14 different countries, most frequently in Finland (*n* = 3; 13.1%) and Switzerland (*n* = 3; 13.1%). The aim of the studies was to investigate the function and activities of RRS (*n* = 11; 47.8%), patient outcomes (*n* = 10; 43.5%) and its effectiveness/feasibility and costs (*n* = 2; 8.7%). They mainly used cross‐sectional studies (*n* = 11; 47.8%) and retrospective designs (*n* = 7; 30.4%), less frequently trials (*n* = 3; 13.1%) and prospective studies (*n* = 2; 8.7%).

**TABLE 1:** Characteristics of the included studies (*n* = 23).

Characteristics	*N* (%)	References
Publication years		
2000–2010	4 (17.4)	[18, 19, 20, 21]
2011–2020	13 (56.5)	[10, 22, 23, 24, 25, 26, 27, 28, 29, 30, 31, 32, 33]
2021–2024	6 (26.1)	[34, 35, 36, 37, 38, 39]
Country		
Finland	3 (13.1)	[10, 22, 33]
Switzerland	3 (13.1)	[21, 24, 36]
Belgium	2 (8.7)	[27, 39]
England	2 (8.7)	[19, 20]
France	2 (8.7)	[28, 29]
Germany	2 (8.7)	[21, 35]
Italy	2 (8.7)	[25, 26]
Netherland	2 (8.7)	[30, 31]
Spain	2 (8.7)	[34, 37]
Austria	1 (4.3)	[21]
Cyprus	1 (4.3)	[18]
Denmark	1 (4.3)	[10]
Poland	1 (4.3)	[23]
Portugal	1 (4.3)	[32]
Aim		
To describe function and activities	11 (47.8)	[10, 19, 21, 22, 25, 26, 28, 33, 34, 36, 39]
To explore patients' outcomes	10 (43.5)	[18, 20, 23, 24, 27, 29, 31, 32, 35, 38]
To evaluate effectiveness and feasibility	2 (8.7)	[30, 37]
Design		
Cross‐sectional	11 (47.8)	[10, 19, 21, 25, 26, 28, 30, 33, 34, 36, 39]
Retrospective	7 (30.4)	[22, 23, 24, 29, 32, 37, 38]
Trials	3 (13.1)	[20, 27, 31]
Prospective	2 (8.7)	[18, 35]
Setting (hospitals)		
University or tertiary level	9 (39.1)	[18, 22, 23, 24, 28, 29, 32, 35, 37]
Community and university	6 (26.1)	[18, 21, 30, 31, 33, 38]
Not specified	8 (34.8)	[10, 19, 20, 26, 27, 34, 36, 39]
Sites		
Multicentric	15 (65.2)	[10, 19, 21, 25, 26, 27, 28, 29, 30, 31, 33, 34, 36, 38, 39]
Monocentric	8 (34.8)	[18, 20, 22, 23, 24, 32, 35, 37]
Data collection tool		
Registries	7 (30.4)	[18, 22, 23, 24, 29, 32, 38]
Online questionnaire	6 (26.1)	[25, 28, 30, 34, 36, 39]
Study database	5 (21.7)	[20, 27, 31, 35, 37]
Mail	3 (13.1)	[19, 21, 33]
Telephone	2 (8.7)	[10, 26]
Data collection duration		
≤ 1 month	5 (21.7)	[19, 30, 33, 36, 37]
≤ 1 year	11 (47.8)	[10, 18, 20, 21, 22, 23, 25, 26, 28, 34, 39]
> 1 year	7 (30.4)	[24, 27, 29, 31, 32, 35, 38]

*Note:* Some items are considered in multiple articles.

The studies were conducted at universities/tertiary hospitals (*n* = 9; 39.1%) or in community and university hospitals (*n* = 6; 26.1%), although some studies did not specify where they were conducted (*n* = 8; 34.8%). Fifteen (65.2%) were multi‐centre, while eight (34.8%) were monocentric; only one included three countries, namely Austria, Germany and German Switzerland [21]. A total of 941 hospitals were involved, ranging from two [38] to 197 [25] hospitals in multi‐centre studies, with 180 [38] to 1500 [28] beds. The most used data collection tools were registries (*n* = 7; 30.4%), followed by online questionnaires (*n* = 6; 26.1%). Overall, the studies lasted between 15 days [36] and 5 years [38].

### 4.2 Characteristics of RRS Teams

Overall, the presence or absence of RRS was measured in different hospitals in nine (39.1%) multi‐centre studies. The percentage of hospitals equipped with RRS varied from 31% in 2014 in Finland [33] to 100% in 2021 in Belgium [39]. Different terminologies were used in the studies, with MET (*n* = 6, 26.1%), CAT (*n* = 6; 26.1%) and RRT (*n* = 5; 21.7%) being commonly used (Table 2).

**TABLE 2:** Characteristics of the RRS teams described in the studies (*n* = 23).

Variable	*N* (%)	References
Team		
MET	6 (26.1)	[22, 24, 25, 32, 33, 36]
CAT	6 (26.1)	[10, 18, 21, 33, 35, 39]
RRT	5 (21.7)	[23, 29, 30, 31, 34]
CCO	2 (8.7)	[19, 20]
EMS	1 (4.3)	[38]
Not specified	4 (17.4)	[26, 27, 28, 37]
Availability		
24/7	13 (56.5)	[10, 19, 22, 24, 27, 29, 32, 33, 34, 35, 36, 38, 39]
Variable, during the day	2 (8.7)	[25, 30]
Not specified	6 (26.1)	[18, 21, 23, 26, 28, 31]
Composition		
Nurse	11 (47.8)	[18, 19, 20, 21, 25, 28, 29, 33, 35, 37, 39]
ICU physician	10 (43.5)	[20, 22, 24, 26, 30, 31, 32, 34, 35, 36]
ICU nurse	8 (34.8)	[22, 24, 30, 31, 32, 34, 35, 36]
Physician	8 (34.8)	[10, 21, 25, 28, 29, 33, 35, 39]
Resident	8 (34.8)	[10, 18, 28, 29, 30, 34, 36, 39]
ER physician	2 (8.7)	[26, 36]
Paramedic	2 (8.7)	[38, 39]
Anaesthesiologist	2 (8.7)	[36, 37]
Specialised nurse	2 (8.7)	[10, 38]
Internal medicine physician	1 (4.3)	[36]
Laboratory technician	1 (4.3)	[10]
Medical assistant	1 (4.3)	[10]
Healthcare assistant	1 (4.3)	[10]
Not specified	2 (8.7)	[23, 27]
Staff		
Not exclusively dedicated	6 (26.1)	[24, 25, 26, 34, 35, 39]
Exclusively dedicated	1 (4.3)	[19]
Not specified	16 (69.6)	[10, 18, 20, 21, 22, 23, 27, 28, 29, 30, 31, 32, 33, 36, 37, 38]
Stationing location		
Different units	8 (34.8)	[21, 25, 26, 28, 33, 34, 35, 39]
ICU	7 (30.4)	[22, 24, 29, 30, 31, 32, 36]
Out‐of‐hospital service	1 (4.3)	[38]
Not specified	7 (30.4)	[10, 18, 19, 20, 23, 27, 37]
Activated by		
Ward staff	4 (17.4)	[18, 20, 24, 25]
Physicians or nurses	4 (17.4)	[30, 31, 34, 36]
Exclusively by nurses	2 (8.7)	[22, 38]
Anyone (including patients and family members)	2 (8.7)	[28, 33]
Exclusively by physicians	1 (4.3)	[29]
Not specified	10 (43.5)	[10, 19, 21, 23, 26, 27, 32, 35, 37, 39]
Phone number		
More than one number	6 (26.1)	[25, 26, 28, 29, 34, 39]
Single number different than 2222	2 (8.7)	[18, 38]
Not specified	15 (65.2)	[10, 19, 20, 21, 22, 23, 24, 27, 30, 31, 32, 33, 35, 36, 37]
Activation criteria		
Alteration of specific parameters	9 (39.1)	[22, 24, 28, 29, 30, 32, 33, 34, 36]
Concern/worry of the staff	9 (39.1)	[20, 22, 24, 25, 29, 32, 33, 34, 36]
Multi‐parameter systems	8 (34.8)	[20, 25, 27, 30, 31, 33, 34, 36]
Cardiac arrest	7 (30.4)	[10, 18, 21, 29, 33, 35, 39]
Seizures	3 (13.1)	[24, 29, 32]
MEWS	2 (8.7)	[25, 31]
NEWS	2 (8.7)	[27, 36]
Not specified	5 (21.7)	[19, 23, 26, 37, 38]
Communication tools		
SBAR	5 (21.7)	[27, 29, 30, 31, 34]
Not specified	18 (78.3)	[10, 18, 19, 20, 21, 22, 23, 24, 25, 26, 28, 32, 33, 35, 36, 37, 38, 39]
Activated by		
Medical wards	3 (13.1)	[18, 36, 38]
Surgical wards	2 (8.7)	[18, 36]
Outpatient clinics	1 (4.3)	[38]
Not specified	20 (86.9)	[10, 18, 19, 20, 21, 22, 23, 24, 25, 26, 27, 28, 29, 30, 31, 32, 33, 34, 35, 37]
Activated why		
Cardiac arrest	8 (34.8)	[10, 18, 21, 28, 29, 34, 35, 39]
Alteration of vital parameters	6 (26.1)	[22, 23, 24, 29, 34, 38]
Concern/worry of the staff	4 (17.4)	[23, 24, 29, 34]
Poor clinical conditions	3 (13.1)	[22, 34, 38]
Visits and follow‐up	2 (8.7)	[19, 22]
Support of ward staff	1 (4.3)	[19]
Not specified	10 (43.5)	[20, 25, 26, 27, 30, 31, 32, 33, 36, 37]
Time from activation to arrival		
≤ 5 min	3 (13.1)	[18, 24, 29]
Not specified	20 (86.9)	[10, 19, 20, 21, 22, 23, 25, 26, 27, 28, 30, 31, 32, 33, 34, 35, 36, 37, 38, 39]
Time spent on site		
30 min	3 (13.1)	[22, 24, 32]
Not specified	20 (86.9)	[10, 18, 19, 20, 21, 23, 25, 26, 27, 28, 29, 30, 31, 33, 34, 35, 36, 37, 38, 39]
Main activities performed		
Airway management, ventilation and oxygen therapy	6 (26.1)	[10, 19, 23, 29, 32, 38]
Cardiopulmonary resuscitation	6 (26.1)	[10, 18, 21, 32, 35, 38]
Administration of drugs or fluids	6 (26.1)	[10, 19, 23, 29, 32, 38]
Insertion of peripheral or central venous catheters	4 (17.4)	[10, 23, 29, 32]
Diagnostic procedures	3 (13.1)	[19, 23, 38]
Blood transfusions	3 (13.1)	[19, 29, 32]
Defibrillation, cardioversion or pacing	3 (13.1)	[10, 29, 32]
Consultations	2 (8.7)	[23, 29]
Patient monitoring	1 (4.3)	[23]
DNR decisions	1 (4.3)	[19]
Not specified	13 (56.5)	[20, 24, 25, 26, 27, 28, 30, 31, 33, 34, 36, 37, 39]
Activity registration		
By 76%–100% of hospitals	14 (60.9)	[10, 18, 20, 22, 23, 24, 25, 29, 30, 31, 32, 33, 38, 39]
By 26%–50% of hospitals	2 (8.7)	[34, 36]
Not specified	7 (30.4)	[19, 21, 26, 27, 28, 35, 37]

*Note:* Some items are considered in multiple articles.

Abbreviations: CAT, cardiac arrest team; CCO, critical care outreach; DNR, do not resuscitate; EMS, emergency medical service; ICU, intensive care unit; MET, medica emergency team; MEWS, modified early warning score; NEWS, new early warning score; RRS, rapid response system; RRT, rapid response team; SBAR, situation background assessment recommendation.

Operationally, RRS are mostly available 24/7 (*n* = 15; 65.2%); in the remaining studies, RRS are available in a timeframe (*n* = 2; 8.7%), while six (26.1%) studies did not report the data. The composition described in 21 (91.3%) studies indicated that a physician and a nurse were present in almost all teams, with the exception of CCO, which consisted of nurses only. In a Danish study, CAT were described as consisting of several professionals, including a laboratory technician, a medical assistant and a nursing assistant [10]. In addition, most studies did not specify whether staff were dedicated exclusively to the team (*n* = 16; 69.6%), and when described, staff were predominantly not dedicated exclusively (*n* = 6; 26.1%).

The location of placement, although not specified in some studies (*n* = 7; 30.4%), was mainly the ICU (*n* = 7; 30.4%) and different units (*n* = 8; 34.8%) such as the emergency department and operating theatre.

Although almost half of the studies did not report data (*n* = 10; 43.5%), a range of options emerged as to who was allowed to activate the RRS, with all members of staff (*n* = 4; 17.4%) and physicians or nurses (*n* = 4; 17.4%) being the most common. The number to call to activate the RRT was reported in eight (34.8%) studies and was more than one number in six (26.1%) studies, although data were not reported in several studies (*n* = 15; 65.2%).

Criteria reported for RRS activation in most studies (*n* = 18; 78.3%) were changes in specific parameters (*n* = 9; 39.1%), staff concerns/worries (*n* = 9; 39.1%), multiparametric system data (*n* = 8; 34.8%) and specific scores (i.e., Modified Early Warning Score [MEWS] and National Early Warning Score [NEWS], both *n* = 2; 8.7%). However, the recommended tools for communication between staff and the RRS were specified in only five (21.8%) studies where the Situation, Background, Assessment, Recommendation (SBAR) was reported, while they were not specified in the remainder (*n* = 18; 78.2%).

The units in which RRS were most called are described in three (13.1%) studies, mostly as medical wards. CA (*n* = 9; 39.1%) and change in vital signs (*n* = 6; 26.1%) were the main reasons, although several studies did not provide data (*n* = 10; 43.5%).

The timing of RRT interventions was documented as the time taken for the team to arrive in five (21.7%) studies and ranged from 1.6 (1.3–1.8) minutes [18] to 5 (5–10) minutes [24, 29]. The time spent on site was described in three (13.1%) studies and ranged from 25 (15–30) [24] to 35 (20–50) minutes [32]. One study [18] also described the time between arrival and first shock for ventricular fibrillation or pulseless ventricular tachycardia, the time to administration of the first dose of epinephrine for pulseless electrical activity or asystole, and to airway management: 1.5 (0.87–2.1) minutes, 2.7 (2.2–3.2) minutes and 6.4 (4.4–8.4) minutes, respectively. However, 18 (78.3%) studies did not specify the time required.

The most important activities performed in the field were described in 10 (43.5%) studies as airway management, ventilation and oxygen therapy, cardiopulmonary resuscitation and fluid/medication management. Sixteen (69.6%) studies reported that the activities performed by the RRS were usually recorded in specific documentation.

### 4.3 Patient Outcomes

Twelve (52.2%) studies reported patient outcomes mainly related to mortality rates, CA and transfers, documenting their variation when patients were cared for by the RRS team (Table 3).

**TABLE 3:** Patients' outcomes reported in the studies (*n* = 23).

Outcomes	*N* (%)	References
Mortality		
Mortality rate	3 (13.1%)	[27, 35, 38]
Reduction in mortality rate	6 (26.1%)	[20, 23, 29, 31, 32, 37]
Cardiac arrests		
Cardiac arrest rate	2 (8.7%)	[18, 27]
Reduction in cardiac arrest rate	5 (21.7%)	[23, 24, 29, 31, 37]
Transfers		
Transfers to ICU	4 (17.4%)	[22, 24, 27, 29]
Reduction in transfers to ICU	3 (13.1%)	[23, 31, 37]
Transfers to ED	1 (4.3%)	[38]

Abbreviations: ED, emergency department; ICU, intensive care unit.

Mortality rates were assessed by nine (39.1%) studies [20, 23, 27, 29, 31, 32, 35, 37, 38]; their reduction ranged from −1.5 deaths per 1000 hospitalisations [23] to −5 deaths per 1000 discharges [29]. Various measures have been used to assess mortality as a reduction in the number of deaths per 1000 hospitalisations [23] or per 1000 discharges [29], as a reduction in the OR [20, 31], a percentage reduction [32] or only writing that mortality was decreased [37].

Seven (30.4%) studies described the rate of CAs [18, 23, 24, 27, 29, 31, 37], reporting a reduction from 0.72 CAs per 1000 admissions [31] to −50% [24]. In one study [27], no statistically significant differences in mortality were found when comparing data before and after the introduction of RRS.

Transfer of patients to another unit was reported by eight (34.8%) studies [22, 23, 24, 27, 29, 31, 37, 38], and continuation of treatments was performed in the ICU in almost all studies. One (4.3%) study [27] considered unexpected deaths (1.5 vs. 0.7/1000 admissions, OR 0.82, 95% CI 0.34–1.95).

## 5 Discussion

### 5.1 Characteristics of the Studies

This scoping review has mapped RRS across Europe and provided an overview of the state of research and practice in this area. In doing so, it complements the available studies conducted in Australia [8], where these models of care originated, and in the USA, where they are widely used [12]. In the last 10 years, research has experienced an interesting upswing, as shown by the number of publications [22, 23], which reflect international trends [40]. The publication of the European Resuscitation Council (ERC) guidelines [41] and the International Recommendations of the International Liaison Committee on Resuscitation (ILCOR) [6], suggesting the implementation of RRS for the treatment of in‐hospital CAs, may have sparked a renewed interest in this topic. However, studies have been conducted in 14 out of 44 European countries, spread across the different regions, except for Eastern Europe, where only one study conducted in Poland [23] is available, suggesting that a more comprehensive approach is needed to identify the main characteristics of the team in the East.

The available studies mainly aim to describe the main characteristics of RRS, mostly using descriptive approaches, thus providing elements on team structure and process [25, 26], while a few examine outcomes [18, 23]. On the one hand, the low level of interest in outcomes may indicate that the effects of RRS are not considered to need investigation given the evidence to date [9]. Conversely, the predominant interest in organisational characteristics may have been triggered by the gaps in the field, the different solutions implemented, and the need to describe these in practical terms and make comparisons between hospitals in a kind of benchmarking approach. This is also reflected in the profile of the published studies, which were mainly multi‐centre and national in scope. However, examining the differences in outcomes according to the different RRS organisations is crucial to identify the best model; therefore, the next generation of studies should combine descriptive intent with patient impact in a more comprehensive approach across countries.

Three main sources of data have emerged, namely questionnaires (postal, email or telephone), register‐based and clinical data. No commonalities have emerged between studies in the instruments used, including registries, which are mostly conducted at the local level [42]: a homogeneous approach could be helpful in future research in expanding this area of research. The Utstein guidelines [43], which specify standardised methods for measuring and reporting variables relevant to in‐hospital and out‐of‐hospital CA to facilitate and structure research and publication on resuscitation, do not appear to have been followed.

### 5.2 Characteristics of RRS Teams

Overall, the characteristics documented to date suggest great diversity in the field of RRS in Europe, with non‐homogeneous structures and processes, although the often‐unspecified data call for greater harmonisation of this research. It has been shown that the availability of RRSs in hospitals has increased (e.g., from 31% to 100%), although it is not mandatory in all countries or, when recommended, is still inconsistently implemented, for example, in Italy [44] and Finland [45]. In 2011, the Australian Commission on Safety and Quality in Health Care published a set of 10 standards recommending the implementation of RRS [46], which have been mandatorily assessed in the accreditation of public and private acute care hospitals since 2013. As a result, the implementation of RRSs increased from 66% to 85% of hospitals [46]. Guidelines and recommendations could also harmonise the different terms in Europe: although a clear distinction was made between METs and RRTs [4], studies used different terminologies to identify the same type of team [47]. In addition, some studies use the general term RRS without specifying the type of team characterising the efferent component [26, 27, 28, 37].

When reported, RRS are available 24/7; this may impact patients' access to the best care (e.g., during the night shift [1]) and raise ethical issues when not available. Limited availability may affect nurses and increase concerns about providing the best care, especially in medical departments where the need for RRS is greatest according to previous studies [48] and nurse‐to‐patient ratios are poor. Although the optimal composition of RRS is not known [7], a different picture has emerged across studies in terms of team members, possibly reflecting available resources and profiles [10]. Only in the UK are physicians not represented in CCO, as they are led by nurses [19, 20], while in the Finnish EMS there are two paramedics, one of whom is advanced, that is, a nurse specialised in intensive care [38]. The expansion of nurses' competences in Anglo‐Saxon and Scandinavian countries, which are increasing their autonomy [49], may have influenced the composition of the RRS. However, given the diversity of healthcare professions involved, it is strongly recommended that the impact on patients be measured to identify any differences.

The question of whether staff are solely responsible for the RRS should also be investigated further: if staff are not RRS‐only, this may have various effects on the rest of the staff due to the increased workload of colleagues who have to cover for RRS members, which may also have an impact on patients [50]. In addition, the performance of the team may be jeopardised as they are forced to interrupt the care of their own patients, hand them over to another colleague, and then care for another patient in a different unit as a member of the RRS [51]. This can be exacerbated if the ward location is not close to their own ward. To avoid this problem, it has been suggested that they should not be involved in the direct care of patients if they are members of the RRS [29, 52].

The staff authorised to activate the team are mainly those of the wards in some studies [24, 30]; in another, only physicians or nurses [22]. Only in a few studies could relatives or patients also activate the RRS [28, 33], although this option has been recommended [7]. Although concerns have been raised about the increased workload of RRS staff, activations by patients or family members are uncommon and usually occur when there is a breakdown in communication between the ward team and them [7]. Family members can provide information about the patient's baseline mental and/or functional status and recognise changes at an early stage [53].

The telephone number for activating the RRS also varies both across and within countries, as documented by Haegdorens et al. [27], as different telephone numbers were used in almost all (*n* = 18, 94.7%) Belgian hospitals. Overall, only two (8.7%) studies documented the use of a standardised number, which differs from the proposed 2222 [41]. The use of a Europe‐wide standardised number could reduce the occurrence of call delays and confusion among staff in different hospitals [54].

The available guidelines recommend the use of clear RRS activation criteria that are shared by all staff [7]. Regardless of whether a single‐ or multi‐parameter alert score is used, it is important to consider staff concern about the patient's clinical condition, which may occur even before the change in vital signs, as a criterion for team activation [55]. Concern and changes in parameters were reported as criteria for RRS activation in most studies [22], which is consistent with the review by Feroz Ali et al. [56]. The reasons for RRS activation are related to the proximity of the activities performed, ranging from cardiopulmonary resuscitation to invasive procedures (e.g., orotracheal intubation). The lack of standardisation of the means of communication used when calling the RRS, as well as the documentation of the interventions performed and the results/follow‐ups, requires further efforts [57].

The reported response time of the RRS is between 1.6 [18] and 5 min [24, 29]. This is consistent with the study by Yang et al. [58] from China, in which the average arrival time was 2.4 (0.1) minutes, and the study by Almeida et al. [59] from Brazil, in which it was less than 5 min. The team stayed on site between 25 [24] and 35 min [32], which is consistent with the study conducted in the USA by Wang et al. [60], in which all teams stayed on site for more than 20 min.

### 5.3 Patient Outcomes

Three main outcomes have been documented to date concerning the in‐hospital mortality, the CAs occurrence and the transfer to intensive care following the RRS treatment. Different metrics have been used, limiting the comparison of the data between studies confirming a well‐documented issue [61] and suggesting that more harmonisation in this research field is required. Previous reviews [62, 63] found a reduction in mortality in 60% of trials, while the review by Zhang et al. [64] described a minimal difference (48.1% of the studies found a reduction in mortality). The main intent of RRS is to reduce CAs [64]: in our review, the reduction in CAs ranged from 21.4% [23] to 50% [24], which is consistent with the study by Viana et al. [65] in Brazil, according to which the reduction was 40.5%, and with the international meta‐analysis by Rocha et al. [63] and the international systematic review by Zhang et al. [64], with 81.8% and 56.9% of studies, respectively.

Unplanned transfers to the ICU after RRS intervention, ranging between 20.7% [22] and 72% [38], are consistent with the international meta‐analysis by Maharaj et al. [2] and the narrative review by Lyons et al. [66], in which they were 33% and 30%, respectively, but are higher than in the study conducted in South Korea by Kim et al. [67], in which it is 7.4%. However, it should be noted that 20.7% were due to METs [22] and 72% to EMS [38], the activation of which occurred mainly in the event of CA, so the high rate of transferred patients is to be expected.

Regarding the changes in ICU transfers after the introduction of RRS in hospitals, they vary between −29.2% [23] and +15.5% [29]. In the systematic review by Zhang et al. [64], 46.7% of studies found a decrease in ICU transfers, 40% found no difference, and 13.3% found an increase. If patients with clinical deterioration are recognised early, unplanned ICU admissions may increase as they are more likely to be transferred from the general ward to the ICU. Conversely, effective team activation could reduce the rate of ICU admissions by assessing and treating patients with clinical deterioration immediately on the ward. In addition, ICU admission rates depend mainly on bed capacity, the ratio of beds to ICUs and inclusion criteria [64].

## 6 Implications for Practice and/or Further Research

Future research should investigate outcome variations between different types of RRS. Guidelines standardising the minimum data set in this area, by also introducing registries with a more consistent metric regarding patient outcomes, should be followed to ensure consistency between practices and studies.

## 7 Limitations

This scoping review has several limitations. Although four databases were systematically searched, some publications may not have been found because they are not indexed in the databases, were published as grey literature or are written in languages other than English. Data extraction was carefully performed by two reviewers; however, given the large amount of unspecified data, some authors could have been contacted to complete the data but were not.

## 8 Conclusion

This scoping review mapped RRS across Europe and provides an overview of research and practice in this area. Numerous studies have emerged over the last decade, which are mainly descriptive and multi‐centre, with Eastern European countries under‐represented. Most studies focus on organisational characteristics, particularly the afferent and efferent limbs of the RRS, rather than patient outcomes. Heterogeneous patterns of RRS organisation have emerged, including terminologies, team composition, availability, activation systems and activities. Data collection varied across studies, with several ad hoc instruments being used, resulting in unspecified data in some studies. Specific guidelines are needed to promote and improve RRS and to standardise research in this field.

## Author Contributions

**Sara Zamò:** conceptualisation, investigation, formal analysis, data curation, writing – original draft. **Federico Fonda:** formal analysis, data curation, writing – original draft. **Alvisa Palese:** conceptualisation, formal analysis, methodology, validation, writing – review and editing, supervision, project administration. **Alessandro Galazzi:** conceptualisation, investigation, formal analysis, methodology, validation, writing – review and editing, supervision. All authors critically revised the content of the manuscript, read and approved the final version.

## Ethics Statement

As this scoping review is based on the analysis of previously published literature, ethical approval was not required.

## Conflicts of Interest

The authors declare no conflicts of interest.

## Supporting information


**Table S1:** Preferred Reporting Items for Systematic reviews and Meta‐Analyses extension for Scoping Reviews (PRISMA‐ScR) checklist.
**Table S2:** Database search strings.
**Table S3:** Characteristics of the included studies.

## Data Availability

Data sharing not applicable to this article as no datasets were generated or analysed during the current study.
