# Supplementary material for: What Evidence is Available on Rapid Response Systems Across Europe? Findings From a Scoping Review
Source: Nurs Crit Care. 2025 Oct 23;30(6):e70217. doi: 10.1111/nicc.70217 (PMC12550357; doi:10.1111/nicc.70217)
Supplement: Supplementary file 1 — Table S1: Preferred Reporting Items for Systematic reviews and Meta‐Analyses extension for Scoping Reviews (PRISMA‐ScR) checklist. Table S2: Database search strings. Table S3: Characteristics of the included studies. [file NICC-30-0-s001.doc]

**Table S1.** Preferred Reporting Items for Systematic reviews and Meta-Analyses extension for Scoping Reviews (PRISMA-ScR) checklist^

| **SECTION** | **ITEM** | **PRISMA-ScR CHECKLIST ITEM** | **REPORTED ON MANUSCRIPT SECTION** |
| --- | --- | --- | --- |
| **TITLE** | | | |
| Title | 1 | Identify the report as a scoping review. | Title |
| **ABSTRACT** | | | |
| Structured summary | 2 | Provide a structured summary that includes (as applicable): background, objectives, eligibility criteria, sources of evidence, charting methods, results, and conclusions that relate to the review questions and objectives. | Abstract |
| **INTRODUCTION** | | | |
| Rationale | 3 | Describe the rationale for the review in the context of what is already known. Explain why the review questions/objectives lend themselves to a scoping review approach. | Background |
| Objectives | 4 | Provide an explicit statement of the questions and objectives being addressed with reference to their key elements (e.g., population or participants, concepts, and context) or other relevant key elements used to conceptualize the review questions and/or objectives. | Background |
| **METHODS** | | | |
| Protocol and registration | 5 | Indicate whether a review protocol exists; state if and where it can be accessed (e.g., a Web address); and if available, provide registration information, including the registration number. | Design |
| Eligibility criteria | 6 | Specify characteristics of the sources of evidence used as eligibility criteria (e.g., years considered, language, and publication status), and provide a rationale. | Research question, Inclusion and Exclusion Criteria |
| Information sources^*^ | 7 | Describe all information sources in the search (e.g., databases with dates of coverage and contact with authors to identify additional sources), as well as the date the most recent search was executed. | Search strategy, Supplementary Table 2 |
| Search | 8 | Present the full electronic search strategy for at least 1 database, including any limits used, such that it could be repeated. | Search strategy, Supplementary Table 2 |
| Selection of sources of evidence^†^ | 9 | State the process for selecting sources of evidence (i.e., screening and eligibility) included in the scoping review. | Selection of studies |
| Data charting process^‡^ | 10 | Describe the methods of charting data from the included sources of evidence (e.g., calibrated forms or forms that have been tested by the team before their use, and whether data charting was done independently or in duplicate) and any processes for obtaining and confirming data from investigators. | Data Extraction and Synthesis |
| Data items | 11 | List and define all variables for which data were sought and any assumptions and simplifications made. | Data Extraction and Synthesis |
| Critical appraisal of individual sources of evidence^§^ | 12 | If done, provide a rationale for conducting a critical appraisal of included sources of evidence; describe the methods used and how this information was used in any data synthesis (if appropriate). | Not performed |
| Synthesis of results | 13 | Describe the methods of handling and summarizing the data that were charted. | Data Extraction and Synthesis |
| **RESULTS** | | | |
| Selection of sources of evidence | 14 | Give numbers of sources of evidence screened, assessed for eligibility, and included in the review, with reasons for exclusions at each stage, ideally using a flow diagram. | Selection of studies, Figure 1 |
| Characteristics of sources of evidence | 15 | For each source of evidence, present characteristics for which data were charted and provide the citations. | Results, Table 1, Table 2, Supplementary Table 3 |
| Critical appraisal within sources of evidence | 16 | If done, present data on critical appraisal of included sources of evidence (see item 12). | Not performed |
| Results of individual sources of evidence | 17 | For each included source of evidence, present the relevant data that were charted that relate to the review questions and objectives. | Results, Table 1, Table 2, Supplementary Table 3 |
| Synthesis of results | 18 | Summarize and/or present the charting results as they relate to the review questions and objectives. | Results, Table 1, Table 2, Supplementary Table 3 |
| **DISCUSSION** | | | |
| Summary of evidence | 19 | Summarize the main results (including an overview of concepts, themes, and types of evidence available), link to the review questions and objectives, and consider the relevance to key groups. | Discussion |
| Limitations | 20 | Discuss the limitations of the scoping review process. | Limitations |
| Conclusions | 21 | Provide a general interpretation of the results with respect to the review questions and objectives, as well as potential implications and/or next steps. | Conclusions |
| **FUNDING** | | | |
| Funding | 22 | Describe sources of funding for the included sources of evidence, as well as sources of funding for the scoping review. Describe the role of the funders of the scoping review. | No funding to declare |

Abbreviations: PRISMA-ScR, Preferred Reporting Items for Systematic reviews and Meta-Analyses extension for Scoping Reviews.

^ Tricco et al. PRISMA Extension for Scoping Reviews (PRISMA-ScR): Checklist and Explanation. Ann Intern Med. 2018;169(7):467-473.

* Where *sources of evidence* (see second footnote) are compiled from, such as bibliographic databases, social media platforms, and Web sites.

† A more inclusive/heterogeneous term used to account for the different types of evidence or data sources (e.g., quantitative and/or qualitative research, expert opinion, and policy documents) that may be eligible in a scoping review as opposed to only studies. This is not to be confused with *information sources* (see first footnote).

‡ The frameworks by Arksey and O’Malley (2015), Levac and colleagues (2010), and the Joanna Briggs Institute guidance (Peters et al., 2015; Peters et al., 2017) refer to the process of data extraction in a scoping review as data charting*.*

§ The process of systematically examining research evidence to assess its validity, results, and relevance before using it to inform a decision. This term is used for items 12 and 19 instead of "risk of bias" (which is more applicable to systematic reviews of interventions) to include and acknowledge the various sources of evidence that may be used in a scoping review (e.g., quantitative and/or qualitative research, expert opinion, and policy document).

**Table S2**. Database search strings

| **Database** | **Data** | **Query** | **Filters** |
| --- | --- | --- | --- |
| CINAHL | On 5 August 2024  769 articles | “hospital rapid response team” [All Text] OR "rapid response system” [All Text] OR "rapid response team” [All Text] OR "medical emergency team" [All Text] OR “outreach critical team” [All Text] OR “cardiac arrest team” [All Text] | Excluded: Reviews, Systematic Reviews, meta-analysis, books, book chapters, editorials  Limited to: English language |
| Cochrane Library | On 5 August 2024  191 articles | “hospital rapid response team” [Title Abstract Keyword] OR "rapid response system” [Title Abstract Keyword] OR "rapid response team” [Title Abstract Keyword] OR "medical emergency team" [Title Abstract Keyword] OR “outreach critical team” [Title Abstract Keyword] OR “cardiac arrest team” [Title Abstract Keyword] | Limited to: Trials, English language |
| PubMed | On 5 August 2024  665 articles | “hospital rapid response team” [MeSH Terms] OR "rapid response system” [All fields] OR "rapid response team" [All fields] OR "medical emergency team" [All fields] OR outreach critical team [All fields] OR “cardiac arrest team” [All fields] | Excluded: Reviews, Systematic reviews, meta-analysis, editorials, books  Limited to: English language |
| Scopus | On 5 August 2024  7044 articles | “hospital rapid response team” [All fields] OR "rapid response system” [All fields] OR "rapid response team” [All fields] OR "medical emergency team" [All fields] OR “outreach critical team” [All fields] OR “cardiac arrest team” [All fields] | Excluded: review, editorial, book, book chapter, conference, conference review, retracted  Limited to: English language, Medicine, Nursing, Health Professions |

**Legend**: CINAHL, Cumulative Index to Nursing and Allied Health Literature

**Table S3**. Characteristics of the included studies.

| ***Author(s)***  ***Publication year***  ***Journal*** | ***Aim*** | ***Design***  ***Data collection period***  ***Setting (number of beds)***  ***Country*** | ***Team*** | ***Availability***  ***Composition***  ***Stationing location***  ***Dedicated staff*** | ***Activated by***  ***Phone number***  ***Activation criteria***  ***Communication tools*** | ***Activated when***  ***Activated why***  ***Activated where***  ***Timing*** | ***Main activities***  ***Activity registration*** | ***Patient outcomes*** |
| --- | --- | --- | --- | --- | --- | --- | --- | --- |
| Azimirad et al.  2016  International Journal of Caring Sciences | To analyze MET function | Retrospective (register-based)  2013-2014  1 university hospital (671 beds)  Finland | MET | 24/7  1 ICU physician and 1 or 2 ICU nurses depending on the intervention  ICU  ND | Ward nurses  ND  sBP< 90, 140<HR<40, 30<RR<8 o 25<RR<11, SpO2 <90, nurses concern  ND | Evening shift (35.2%), on Friday (16.6%), in the months of May (12.1%) and November (10.6%)  Other reasons (e.g MET visits and follow-up) (35.3%), poor general conditions (33.1%), SpO_2_ < 90 (27.8%) and loss of consciousness (22.4%)  ND  The time taken by nurse 1 for MET calls was 31.7 (SD 16.7) minutes; for nurse 2, it was 30.2 (SD 15.5) minutes | MET visits and follow-up  Yes | 20.7% of patients were transferred to the ICU, 68.1% stayed in the original ward or were transferred to another ward. |
| Clemente Vivancos et al.  2022  International Journal of  Environmental Research and Public Health | To describe and analyze the organization and care process for patients at risk of clinical deterioration outside of the ICU | Cross-sectional (online survey)  07/2020 – 01/2021  62 hospitals with an ICU (ND)  Spain | RRT | Available in 51.6% of the hospitals. 24/7 (92%)  An ICU physician (95.5%), an ICU resident (53.2%), an ICU nurse (24.2%), and a nurse dedicated full-time to the RRT in the ICU (3.2%)  ICU (97%), ED (1.5%) or a combination (Anaesthesia, Cardiology and Internal Medicine) (1.5%)  3.2% have a dedicated nurse | Only doctors and nurses (55%), all healthcare professionals (39%), nurses, only if responsible doctors are not available (5%), only doctors (3%)  2222 (1.8%)  Changes in vital signs (85.5%), signs of clinical deterioration (82.3%), clinical concern (56.5%). EWS (10.4%). Measurement of vital signs compatible with NEWS 2 (22%)  SBAR (6.5%) | ND  Risk and/or early signs of clinical deterioration, established signs of instability, moments prior to cardiac arrest, established cardiac arrest, changes in vital signs, signs of clinical deterioration, clinical concern, syndromic presentation, laboratory/blood test abnormalities, blood test abnormalities without changes in vital signs  ND  ND | ND  Utstein-style register (27.4%), number of cardiac arrests (40.3%), number of activations and reasons for activation (30.6%) and results of the intervention (43.5%) | ND |
| Czempik et al.  2019  Anaesthesiology Intensive Therapy | To analyze the interventions and effects of the implementation of the RRT | Retrospective (register-based)  01/10/2018 - 30/01/2019  1 university hospital (ND)  Poland | RRT | ND  ND  ND  ND | ND  ND  ND  ND | ND  Respiratory problems (46%), circulation problems (24%), neurological problems (9%), staff concerns (6%), and others (15%)  ND  ND | Assessment of respiratory efficiency, pharmacological treatment (titration or continuation of antibiotic therapy, inotropes, fluids, mucolytics, anticoagulants, diuretics, opioids, NSAIDs, antiarrhythmics), arterial ABG, endotracheal intubation, modification of ventilation parameters, bronchial suctioning, oxygen therapy, central venous catheter/PICC, alveolar recruitment, extubation, chest X-ray, additional blood tests, monitoring of vital signs, specialist consultations, EKG.  Yes | Reduction in the number of sudden cardiac arrests (-21.4%), reduction in mortality (-1.5 deaths per 1000 admissions), reduction in transfers to ICU (-29.2%). |
| Eftychiou et al.  2009  Hellenic Journal of Cardiology | To assess the frequency of cardiac arrests, the outcomes of resuscitation, and the associations between survival rates and patient characteristics; evaluate the effectiveness of the CAT and identify improvement strategies | Prospective (register-based)  01/2007 - 12/2007  1 tertiary hospital (400 beds)  Cyprus | CAT | ND  1 cardiology resident (team leader), 1 ICU resident, 1 general medicine resident, and 1 nurse, all ALS providers  ND  ND | Ward staff  3333  Cardiac arrest  ND | Between 0:00 and 7:59 (39.7%), between 8:00 and 15:59 (28.6%) and between 16:00 and 23:59 (31.7%)  Cardiac arrest      Coronary care unit (20.3%), cardiology (7.2%), internal medicine (20.3%), ICU (13%), ED (13%), angiothroacic ward (10.1%), surgical ward (5.8%), nephrology (4.3%), orthopedic ward (2.9%), urology (1.4%) and neurosurgery ward (1.4%)  The emergency team arrived within 1.6 minutes (CI 95%: 1.31-1.79), the first shock in VF/VT was delivered within 1.5 minutes (CI 95%: 0.87-2.1) and the first adrenaline dose in cases of asystole/PEA was given within 2.7 minutes (CI 95%: 2.2-3.2). The airway was secured within 6.4 minutes (CI 95%: 4.4-8.4). The time intervals refer to the time from the emergency call | ALS  Yes | Return of spontaneous circulation in 52% of cardiac arrests (90% VF/pulseless VT and 46% asystole/PEA). 17.8% of patients were discharged alive, 82.2% died in hospital |
| Etter et al.  2014  The European Journal of Medical Science | To review the preceding factors, patient characteristics, process parameters and their correlation to patient outcomes of MET calls since the introduction of the team. | Retrospective (register-based)  19/10/2009 - 31/12/2013  1 university hospital (960 beds)  Switzerland | MET | 24/7  1 ICU physician and 1 ICU nurse  ICU  No, they work in the ICU | Any healthcare professional  ND  Threatened airway, 6>RR>36, SpO2<90 despite supplementary oxygen, sBP<90, 40>HR>140, capillary refill time > 3sec, GCS<13 or decrease by ≥2 points, repeated or prolonged seizures, staff concern  ND | Day shift (08:00–16:00) (40%), late shift (16:00–00:00) (40%), and night shift (00:00–08:00) (28%)  Staff concern (28%), alteration of vital signs (58%), including alterations in SpO2 (40%), airway compromise (3.5%), low GCS score (9%), seizures (1.3%)  ND  The median time delay from the alarm to the arrival of the MET on scene was 5 (5-10) minutes and the median time on scene was 25 (15-30) minutes | ND  Yes | Increase in MET calls, from 5.2 to 16.5 per 1000 hospital admissions (p < 0.0001), decrease in cardiac arrest calls in the MET perimeter from 1.6 in 2008 to 0.8 per 1000 admissions (p = 0.014). In 49% MET calls the patient was stabilised on the original ward, a transfer to the ICU or an intermediate care unit was necessary in 40% and 11% cases respectively |
| Galazzi et al.  2020  Acta Biomedica for Health Professions | To describe MET implementation | Cross-sectional (online survey)  27/07/2018 – 10/01/2019  197 hospitals: 79.2% non-university and 20.8% university with ICU service (72.6% < 500 beds, 16.2% 500 - 999, 11.2% > 1000)  Italy | MET | Available in 59.9% of hospitals. 24/7 (84.8%), daytime only 7/7 (4.3%), nighttime only 7/7 (1.7%), always on weekdays - 5/7 (2.5%), daytime only on weekdays - 5/7 (2.5%), nighttime only on weekdays - 5/7 (1.7%), only on holidays (2.5%)  1 physician e 1 nurse  ICU or ED  Dedicated ICU physician (32.2%), non-dedicated ICU physician (67.8%), dedicated nurse (30.5%), non-dedicated nurse (69.5%) | Ward staff  Difference between hospitals  None/subjective evaluation (34.7%), MET (29.7%), Patient At Risk Team (1.7%), MEWS (22.9%), other systems (11.0%).  ND | ND  ND  ND  ND | ND  No (22.9%), yes (77.1%), of which 62% in a specific MET medical record | ND |
| Giusti et al.  2017  Resuscitation | To describe the phone numbers in use to contact the RRS | Cross-sectional (telephone survey)  15/01/2019 – 15/03/2019  152 public hospitals (ND)  Italy | RRS | ND  1 physician working in the ICU (77%), in the ED (6.6%), in a high-intensity medicine unit (1.3%)  ICU or ED  Non dedicated physician 89.5% | ND  2222 in 2.6% of hospitals  ND  ND | ND  ND  ND  ND | ND  ND | ND |
| Haegdorens et al.  2018  Resuscitation | To evaluate the effect of a RRS on the incidence of sudden death, cardiac arrest with cardiopulmonary resuscitation, and unplanned ICU admissions | RCT  10/2013 –  05/2015  7 hospitals (ND)  Belgium | RRS | 24/7  ND  ND  ND | ND  ND  NEWS  SBAR | ND  ND  ND  ND | ND  ND | No significant difference between the control group and the intervention group in rates of unexpected deaths (1.5 vs 0.7/1000, OR 0.82, CI 95% 0.34–1.95), cardiac arrest rates (1.3 vs 1.0/1000, OR 0.71, CI 95% 0.33–1.52) or unplanned ICU admissions (6.5 vs 10.3/1000, OR 1.23, CI 95% 0.91–1.65) |
| Haschemi et al.  2021  Scientific Reports | To compare load-and-go and stay-and treat CATs | Prospective (before-after study)  05/2014 – 04/2018  1 university hospital (ND)  Germany | CAT | 24/7  Load-and-go: 1 ward physician and 1 ward nurse; stay-and treat: ICU physician and ICU nurse  Load-and-go ward; stay-and treat: ICU  No | ND  ND  Cardiac arrest  ND | ND  Cardiac arrest  ND  ND | Load-and-go: BLS and transfer to ICU  Stay-and-treat: ACLS in the ward and transfer to ICU  ND | No difference in survival at discharge between the load-and-go group (33%) and the stay-and-treat group (35%) (p = 0.758). 22% of patients in the load-and-go group compared to 21% in the stay-and-treat group were discharged with good neurological outcomes (p = 0.857) |
| Jouffroy et al.  2018  Anesthesia Critical Care and Pain Medicine | To describe the process and organization of hospital emergencies | Cross-sectional (online survey)  2017  38 public university hospitals (medicine, surgery and obstetrics beds were 956 [450–1500])  France | RRS | Available in 95% of hospitals: 76% for all emergencies, 21% for cardiac arrest only  1 physician (95%), 1 resident (79%), and 1 nurse (84%)  Pre-hospital emergency medical system (29%), ED (16%), and ICU (13%)  ND | Anyone (e.g. staff, family)  Dedicated phone number in 95% of hospitals, the same for cardiac arrest or other emergencies in 84%  Predefined vital signs criteria  (32%) or based on locally developed experience  (21%)  ND | ND  Any emergency, cardiac arrest  ND  ND | ND  ND | ND |
| Jung et al.  2016  Intensive Care Medicine | To evaluate whether the implementation of a physician-led RRT is associated with a decrease in patient mortality | Retrospective (register-based)  07/2010 - 12/2013  4 university hospitals (ND)  France | RRT | 24/7  A physician, a resident, and a nurse only if requested by the physician  ICU  ND | Attending physician (48%), nurse (30%), resident physician (19%), non-medical staff (3%)  37272 or 19198  HR <40 or >140, sBP <80, cardiac arrest, RR <8 or >30, SpO2<90 with O_2_ >6 l/min, respiratory distress in a tracheotomized patient, respiratory arrest, coma or sudden change in level of consciousness, seizure  SBAR | ND  SpO2 <90 (17%), sBP <80 (14%), altered mental status (14%), RR >30 (10%), dyspnea (8%), unspecific clinical concern (6%), HR >140 (6%), cardiac arrest (5%), hemorrhage (4%), seizure (2%), HR <40 (1%), other (13%)  ND  Median time of arrival was 5 (5–10) minutes | Crystalloid infusion  (22%), antibiotics (8%), peripheral line (7%),  intubation (6%), colloid infusion (6%), diuretics (6%), strategy advice only (6%), transfusion (5%), compressions for acute bleeding (4%), nebulizer treatment (4%), vasopressors (4%), antiarrhythmic treatment  (3%), non-invasive ventilation (2%), defibrillation (2%), flumazenil (2%), central venous access (1%), analgesics (1%), antiepileptic drugs (1%), others (8%)  Yes | Decrease in the unexpected mortality rate from 21.9 to 17.4 per 1000 discharges between the pre-RRT period and the post-RRT period (p = 0.002). The implementation was associated with 1.5 lives saved per week. Overall mortality decreased from 39.6 to 34.6 per 1000 discharges between the pre-RRT period and the RRT period in the hospital with RRT (p = 0.012), but did not change in other hospitals. There was a reduction in cardiac arrests during the intervention period (2.6 vs. 1.8 per 1000 admissions; p = 0.07). The rate of ICU admissions increased from 45.8 per 1000 discharges in the pre-RRT period to 52.9 per 1000 discharges in the RRT period (p = 0.002), while the admission rates to the ICU did not change significantly in other hospitals |
| Lauridsen et al.  2015  Resuscitation | To describe CAT composition and activities | Cross-sectional (telephone survey)  13/12/2012 – 30/04/2013  44 public hospitals (ND)  Denmark | CAT | 24/7  The number of team members was 5 (4-6). In large hospitals, it was 6 (5-8); in medium-sized hospitals, it was 5 (4-6); and in small hospitals, it was 3 (3-4).  1 nurse anesthetist (100%), 1 anesthesiologist (82%), 1 healthcare assistant (73%), 1 anesthesiology resident (64%), and 1 medical assistant (20%). Some teams also included a medical laboratory technician.  20% of the teams had a different team composition during nights and weekends. Of these, 78% reduced the number of members by at least one person  ND  ND | ND  ND  Cardiac arrest  ND | ND  Cardiac arrest  ND  ND | Chest compressions, defibrillation, airway handling, medication  Yes | ND |
| Ludikhuize et al.  2011  The Joint Commission Journal on Quality and Patient Safety | To investigate the variation within the components of the RRS and the respondents' opinions on its potential effectiveness and feasibility | Cross-sectional (online survey)  11/2009  63 hospitals of which 9.5% university (ND)  The Netherlands | RRT | Available in 79% of hospitals. 24/7 (89%), from 8:00 to 17:00 (10%), and from 8:00 to 22:00 (1%)  ICU physicians (57%), ICU nurses (57%), depends on the time of the day (11%), exclusively nurse-led (3%), residents (es., general medicine, ED) (14%)  ICU  ND  89% of hospitals are available 24/7, | Physician only (10%), nurses without physician consent (38%), nurses when physicians did not follow the local protocol (48%)  ND  Multiple parameters scoring system  (86%), single parameter systems (11%)  SBAR (67%), Reason-Story-Vital Signs-Plan (14%) | ND  ND  ND  ND | ND  Yes (86%) | ND |
| Ludikhuize et al.  2015  Critical Care Medicine | To describe the effect of the implementation of a RRS on the composite endpoint of cardiac arrest, unplanned ICU admission, or death. | Pragmatic before-after trial  01/04/2009 – 30/11/2011  12 hospitals: 16.7% university hospitals (882– 1000 beds), 66.6% teaching hospitals (359 – 1,070 beds), 16.7% smaller regional hospitals (290 – 325 beds)  The Netherlands | RRT | ND  1 ICU physician and 1 ICU nurse  ICU  ND | Physician or nurse  ND  MEWS >2  SBAR | ND  ND  ND  ND | ND  Yes | Reduction of the composite endpoint of cardiopulmonary arrest, unplanned ICU admission, or death per 1000 admissions in the rapid response team compared to the previous phase (adjusted OR, 0.847; 95% CI, 0.725–0.989; p = 0.036). Reduction in cardiac arrests and in-hospital mortality (OR, 0.607; 95% CI, 0.393–0.937; p = 0.018 and OR, 0.802; 95% CI, 0.644–1.0; p = 0.05, respectively). A downward trend in unplanned ICU admissions was observed (OR, 0.878; 95% CI, 0.755–1.021; p = 0.092), with no difference in disease severity at the time of ICU admission between the periods |
| McDonnel et al.  2007  Journal of Critical Care | To describe the development, introduction, implementation, and current models of CCO | Cross-sectional (postal survey)  02/2005  191 acute care hospitals (ND)  England | CCO | Available in 72.8% of hospitals. 24/7  Service primarily based on nurses  ND  Yes | ND  ND  ND  ND | ND  Timely identification of patients with impending critical illness (85.8%), averting admissions to critical care or ensuring timely admissions to critical care (80.3%), avoiding readmissions to critical care (43.8%), sharing critical care skills with staff  on the wards and in the community (40.5%), enabling discharges from critical care (32%), supporting ward-based care through education at the bedside (31.5%), supporting ward-based staff through formal teaching (19.4%)  ND  ND | Investigations, (e.g. venipuncture, chest x-ray), changes in patient positioning, changes in O_2_ therapy, initiation of noninvasive ventilation, changes in fluid management, initiation of blood/colloid transfusion, initiation of vasoactive infusions, adjustment to medication, adjustment to nutrition, adjustment to pain management, or Initiation of Do-Not-Attempt-Resuscitation decisions  ND | ND |
| Messmer et al.  2024  Minerva Anestesiologica | To analyze the characteristics, prevalence, and organization of METs | Cross-sectional (online survey)  13/11/2021 – 31/11/2021  57 hospitals with an adult ICU (ND)  Switzerland | MET | Available in 44% of hospitals. 24/7 (68%)  ICU physicians (64%), ICU residents (40%), ICU nurses (40%), anesthesiologists (32%), ED physicians (36%), and internal medicine physicians (20%)  ICU  ND | Ward doctor (76%), the nurse in charge (76%), or other health care staff (43%), patients (24%), visitors (16%)  ND  Single abnormal vital sign (80%), clinical concern (68%), NEWS (24.5%)  ND | ND  ND  Medical wards (68%) and surgical wards (64%)  ND | ND  Yes (47%) | ND |
| Muñoz-Rojas et al.  2022  Journal of Clinical Monitoring and Computing | To conduct a cost-effective analysis of the implementation of an RRS | Retrospective (ND)  1/12/2016 – 31/12/2016  1 university hospital (ND)  Spain | RRS | 24/7  1 anesthesiologist and 2 nurses  ND  ND | ND  ND  ND  ND | ND  ND  ND  ND | ND  ND | Reduction in cardiac arrests (-25%), ICU readmissions (-25%), and mortality (-15%) |
| Myrskykari et al.  2024  Australasian Emergency Care | To examine the reasons for calling the EMS and the associated outcomes | Retrospective (register-based)  1/01/2017 – 31/12/2021  1 university hospital (778 beds) e 1 non university hospital (182-337 beds)  Finland | EMS | 24/7  1 advanced level paramedic (emergency-specialized nurse) and 1 basic level paramedic  Out of hospital (3 km from the hospitals)  ND | Nurse (78%), physician (4%)  112  ND  ND | Monday to Friday 8 – 16 (45%). More calls on Tuesdays, in April, but in terms of the distribution of these calls according today or month, there was no  statistically significant difference (p = 0.191 and p = 0.383, respectively)  Cardiac arrest (26%), altered state of consciousness (21%), fainting/syncope (7%), deterioration of general condition (10%), pain (8%), stroke (6%), respiratory failure (6%), seizure (5%), trauma (4%), other (6%)  Internal medicine wards (12%), followed by  ophthalmology outpatient clinics (10%)  ND | Pharmacological therapy (vasopressors, analgesics, sedatives) (33%), IV fluid administration (26%), ECG recording or monitoring (26%), airway management (19%), CPR (14%), supplementary O_2_ (11%), non-invasive ventilation (6%)  Yes | 72% of the patients were transferred to the ED, 100% of death cases were related to a cardiac arrest call, 24% of resuscitated patients were alive 30 days after the event, 44% received a new diagnosis after the EMS visit |
| Priestley et al.  2004  Intensive Care Medicine | To analyze the effects of the introduction of a CCO on mortality and length of stay in an acute care hospital | Pragmatic randomised trial  32 weeks  1 hospital (800 beds)  England | CCO | 24/7  Led by a nurse consultant with a team of experienced nurses. Support from the ICU physician was available as needed, based on the judgment of the support team nurses or the medical staff in the department  ND  ND | Ward staff  ND  Patient-at-Risk, clinical concern  ND | ND  ND  ND  ND | ND  Yes | Reduction in hospital mortality compared to the control group (OR: 0.52 95% CI 0.32–0.85). A possible increase in the length of stay associated with the support was not fully supported by confirmatory and sensitivity analyses |
| Siebig et al.  2009  Deutsches Ärzteblatt International | To understand the composition and training of CATs. | Cross-sectional (postal survey)  10/2006 – 02/2007  35 hospitals in Germany, 7 in Switzerland and Austria both university and non-university hospitals (> 300 beds)  Austria, Germany, German Switzerland | CAT | Available in 91.5% of hospitals  1 (1 - 2) physician and 1.5 (1 - 2) nurses  ICU (80%), operating room (5%), emergency unit (6%)  ND | ND  ND  Cardiac arrest  ND | ND  Cardiac arrest  ND  ND | CPR  ND | ND |
| Silva et al.  2016  Scandinavian Journal of Trauma, Resuscitation and Emergency Medicine | To analyze the activities of the MET and the immediate outcomes for patients | Retrospective (register-based)  01/2012 – 12/2013  1 university hospital (600 beds)  Portugal | MET | 24/7  1 ICU physician and 1 ICU nurse  ICU  ND | ND  ND  Airway threatened (36.8%), clinical concern (31.1%), GCS decreased >2 (25.2%), 90>sBP>200 (21.9%), cardiac arrest (17.5%), 5>RR>36 (9.8%), 40>HR>140 (7.7%), respiratory arrest (7.2%), inappropriate response to treatment (6.2%), repeated/prolonged seizure (3.9%)  ND | Night shift (20:01–8:00) (46%), morning shift (8:01–14:00) (30%), afternoon shift (14:01–20:00) (24%)  ND  ND  The median time the team stayed at the scene was 35 (20–50) minutes. | Fluid challenge (40.6%), bag mask ventilation (37.3%), IV access (11.6%), ventilation (20.1%), endotracheal intubation (15.7%), airway suction (13.6%), CPR (12.3%), oropharyngeal intubation (9.3%), nebulization (6.4%), non-invasive ventilation (3.1%), blood transfusions (2.1%), cardioversion and pacing (2.1%), pharmacological therapy (IV vasopressors [13.6%], IV anesthetics[12.1%], others [11.8%] IV antiarrhythmics [7.7%], IV diuretics [7.7%], IV painkillers [7.5%], bronchodilators [6.2%], steroids [4.4%], IV neuromuscular-blocking [2.3%])  Yes | Reduction in the mortality rate from 4.1% in the three years before implementation to 3.6% in the three years after (p < 0.001) |
| Tirkkonnen et al.  2014  Acta Anaesthesiologica Scandinavica | To describe the characteristics and use of CATs and METs | Cross-sectional (postal survey)  04/2012  51 public hospitals, both university and non-university hospitals with an ICU service  Finland | MET / CAT | CAT available in 57% of hospitals (100% of university hospitals and 84% of non-university hospitals). 24/7 (93%) MET available in 31% of hospitals (100% of university hospitals and 53% of non-university hospitals). 24/7 (87.5%)  CAT 1 physician (100%) and 1 nurse (100%).  MET 1 physician (100%) and 1 nurse (50%) or 2 nurses (50%)  CAT: ICU (62%), operating room (3%), intermediate care unit (11%), ED (25%).  MET: ICU (87.5%), operating room and post-anaesthesia care unit (12.5%)  ND | MET: physicians and nurses 100%, non-medical staff 50%, patients 6.25%, visitors 6.5%  ND  CAT: cardiac arrest  MET: cardiac arrest (93.8%), respiratory arrest (93.8%), clinical concern (93.8%), 5>RR>30 (93.8%), SpO2<90 (93.8%), sBP<90 (93.8%) sBP>200 (6.3%), HR<45 (100%), HR>140 (100%), GCS decrease > 2 points (62.5%), low urine output (12.5%), EWS (18.8%)  ND | ND  ND  ND  ND | ND  CAT yes (80%)  MET yes (93.8%) | ND |
| Verborgh et al.  2021  Acta Anaesthesiologica Belgica | To describe the alert systems, the composition, and the performance of CATs | Cross-sectional (online survey)  08/05/2021 – 31/08/2021  19 hospitals (165 - 950 beds)  Belgium | CAT | Available in 100% of hospitals. 24/7    A specialist physician (92%), in combination with a resident (21%). The nurse is part of the team (100%) and is the only responsible person (10.5%). A paramedic is present as an additional team member (13%). The team composition remains the same at any time of day (83%)  ED (46%) or a combination of ED, ICU and/or anaesthesia (42%) or exclusively ICU (12%)  No, the members of the resuscitation team are determined before the start of the shift (87%) or at the time of the call (13%) | ND  2222 in 10.5% of hospitals. 10.5% of hospitals don’t use a phone number for internal CPR, only a CPR button  Cardiac arrest  ND | ND  Cardiac arrest  ND  ND | ND  Yes (96%) | ND |

**Legend**: ACLS, Advanced Cardiac Life Support; ABG, Arterial Blood Gas; ALS, Advanced Life Support; BLS, Basic Life Support; CAT, Cardiac Arrest Team; CI, Confidence Interval; CCO, Critical Care Outreach; CPR, Cardiopulmonary Resuscitation; CVC, Central Venous Catheter; ECG, Electrocardiogram; ED, Emergency Department; EMS, Emergency Medical Service; EWS, Early Warning Score; GCS, Glasgow Coma Scale; HR, Heart Rate (beat/minute); ICU, Intensive Care Unit; MET, medical emergency team; MEWS, Modified Early Warning Score; ND, Not Determined; NEWS, National Early Warning Score; NSAIDS, Non-Steroidal Anti-Inflammatory Drugs; OR, Odds Ratio; O_2_, Oxygen; PEA, Pulseless Electrical Activity; PICC, Periferically Insterted Central Catheter; RCT, Randomized Controlled Trial; RR, Respiratory Rate (breath/minute); RRS, Rapid Response System; RRT, Rapid Response Team; SBAR, Situation Background Assessment Recommendation; sBP, Systolic Blood Pressure (mmHg); SD, Standard Deviation; SpO2, Peripheral Capillary Oxygen Saturation (%); VF, Ventricular Fibrillation; VT, Ventricular Tachycardia. Data are presented as mean (standard deviation) or median (quartile 1- quartile2).
